# Serum 25-Hydroxyvitamin D Is Decreased with Metabolic Syndrome Following Anterior Cruciate Ligament Reconstruction

**DOI:** 10.3390/nu17152410

**Published:** 2025-07-24

**Authors:** Sonu Bae, Anthony Mantor, Hayden Price, Christopher C. Kaeding, Robert A. Magnussen, David C. Flanigan, Tyler Barker

**Affiliations:** 1 Sports Medicine Research Institute, College of Medicine, The Ohio State University Wexner Medical Center, Columbus, OH 43202, USA; 2 Department of Orthopaedic Surgery, College of Medicine, The Ohio State University Wexner Medical Center, Columbus, OH 43202, USA

**Keywords:** vitamin D, metabolic syndrome, anterior cruciate ligament

## Abstract

**Background/Objectives**: Serum 25-hydroxyvitamin D (25(OH)D) concentrations are decreased with metabolic syndrome (MetSy), and low serum 25(OH)D concentrations are associated with poor outcomes following anterior cruciate ligament (ACL) reconstruction (ACLR). It is unknown whether serum 25(OH)D concentrations are decreased in patients with MetSy following ACLR. The purpose of this study was to investigate whether serum 25(OH)D concentrations are decreased with MetSy following ACLR. **Methods**: This retrospective case–control study consisted of patients (≥18 years) who underwent ACLR. MetSy was defined as meeting any three of the five criteria (cases): (1) body mass index ≥ 30 kg/m^2^, (2) triglycerides ≥ 150 mg/dL, (3) HDL < 40 mg/dL in men and <50 mg/dL in women, (4) systolic blood pressure ≥ 130 mmHg or diastolic blood pressure ≥ 85 mmHg, or (5) estimated (from hemoglobin A1c% [HbA1c]) fasting glucose ≥ 100 mg/dL. Participants without MetSy (meeting <3 criteria) served as controls. The first blood lipid, HbA1c, and 25(OH)D assessed ≥90 d after ACLR were included in this study. **Results**: The final analysis consisted of 219 patients (cases (with MetSy), n = 84; controls (without MetSy), n = 135). Serum 25(OH)D was significantly (*p* < 0.01) decreased (15.8%) in cases (mean [SD]; 25.1 [11.3] ng/mL) compared to controls (29.8 [14.8] ng/mL). An increasing number of MetSy components was associated with a decreased prevalence of vitamin D sufficiency (*p* < 0.01). **Conclusions**: We conclude that serum 25(OH)D concentrations are significantly lower with MetSy. These preliminary findings could provide justification for assessing serum 25(OH)D following ACLR in patients with MetSy and assist with risk stratification.

## 1. Introduction

Metabolic syndrome (MetSy) is a global pandemic characterized by a cluster of interrelated and modifiable metabolic abnormalities and risk factors. These risk factors include elevated blood pressure and fasting glucose, dyslipidemia, and central obesity. Diagnostic criteria vary between different organizations, but in general, abnormal findings for three of the five criteria qualify an individual as having MetSy [1]. The unfortunate consequence of this complex syndrome is illustrated by the increased risk of cardiovascular disease and diabetes mellitus [2], and the rising global prevalence of MetSy [3] represents a major challenge in public health and clinical practice.

Nolan and colleagues [4] provided intriguing evidence illustrating the presence of MetSy and its individual components in young adults (18–30 years). The presence of a single component and a raised MetSy component value at a young age are predictive of developing MetSy [5,6], leading to a lifetime burden of MetSy-related complications or diseases, such as cardiovascular, metabolic, orthopedic, and/or joint diseases [2,7,8]. Knee osteoarthritis is a chronic, degenerative joint disease that can follow a commonly injured and surgically repaired ligament in the knee, the anterior cruciate ligament (ACL) [9]. Knee injuries, such as an ACL rupture, occur throughout the lifespan, but frequently occur in a younger population [10,11]. Considering the prevalence of this injury in younger age groups, patients with an ACL injury and/or who have undergone surgery might provide as an attractive cohort to monitor MetSy and its components in an attempt to mitigate the development of future diseases and disabilities related to this syndrome.

With MetSy and MetSy-related diseases, such as cardiovascular disease and diabetes, circulating vitamin D levels are decreased [12,13,14,15,16,17,18,19]. Vitamin D is a fat-soluble micronutrient, and after multiple hydroxylation reactions, it is converted to a pleiotropic hormone that regulates a range of physiological and biochemical functions. In the circulation, 25-hydroxyvitmain D (25(OH)D) is the metabolite form of vitamin D used to determine vitamin D status [20]. Maintaining a sufficient serum 25(OH)D is necessary to sustain cellular functions, and low circulating concentrations are associated with diverse disabilities, illnesses, and diseases [21]. Serum 25(OH)D concentrations have been found to significantly decrease with increasing components of MetSy in non-vitamin D-deficient elderly adults [17]. Although previous investigations have shown a decrease in serum 25(OH)D concentrations following an ACL injury and surgery [22,23,24,25], there is a gap in our knowledge regarding the association of MetSy and its components with serum 25(OH)D concentrations following ACLR.

The purpose of this study was to test the hypothesis that serum 25(OH)D concentrations are decreased in patients with MetSy following ACLR. Identifying the association between MetSy and serum 25(OH)D after ACLR could achieve the following: (1) reveal modifiable risk factors of future chronic diseases and impairments in a young adult patient population; and (2) provide justification of assessing serum 25(OH)D in patients with MetSy and with components of MetSy. These findings could have potential diagnostic and prognostic implications regarding vitamin D status in relation to metabolic disorders following a common knee injury.

## 2. Materials and Methods

This study consisted of a retrospective case–control design and included patients (male and female, ≥18 y) who underwent arthroscopic ACLR between August 2009 and October 2022 at a single academic institution (The Ohio State University Wexner Medical Center, Columbus, OH, USA). The minimum follow-up was 6 months after ACLR and the last date of data review and extraction from the electronic medical records was 1 June 2023. All data were extracted from electronic medical records and temporally aligned to the date of ACLR. Following study inclusion, patients who underwent ACLR were classified as with (cases) or without (control) MetSy, as defined below. This study complies with the Declaration of Helsinki and was approved with a consent waiver by the Institutional Review Board at The Ohio State University Wexner Medical Center (Columbus, OH, USA).

### 2.1. Subjects

**Figure 1:**
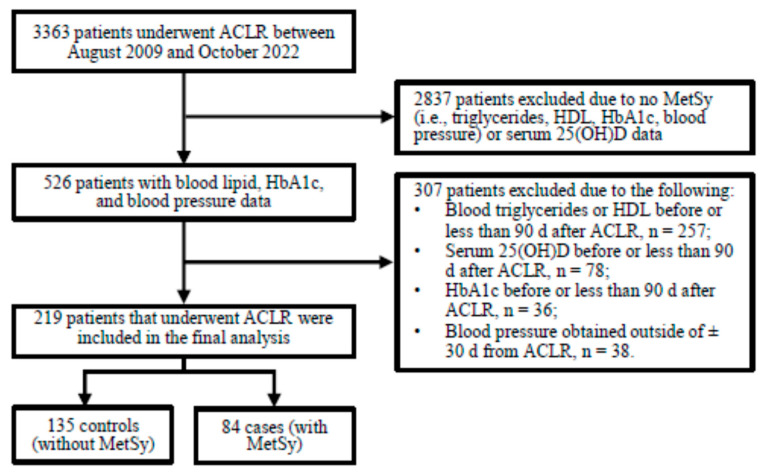
Eligibility screening and flow chart.

Patients were initially identified using Current Procedural Terminology (CPT) codes. Patients who underwent ACLR (CPT code: 29888) were identified and further screened for study inclusion. The inclusion criteria were narrowed to include patients with available blood lipid (i.e., triglycerides and HDL), HbA1c, and serum 25(OH)D data obtained ≥90 d after ACLR, and with blood pressure data obtained within (±)30 d of ACLR (Figure 1).

### 2.2. Clinical Blood Chemistries and Blood Pressures

Demographics, blood chemistries, and corresponding dates were recorded from patients meeting the inclusion criteria. Blood chemistries were performed at The Ohio State University Wexner Medical Center central laboratory (Columbus, OH USA) and conducted as a standard-of-care procedure, separate from ACLR. The first available blood lipid, HbA1c, and the serum 25(OH)D concentration obtained ≥90 d after ACLR were used in this study. It is worth noting that several patients were missing LDL (n = 6), cholesterol/HDL-C (n = 6), and/or non-HDLC data (n = 8), despite having available triglyceride and HDL data needed for the determination of MetSy.

Blood pressure data, preferably obtained the day of ACLR, or, alternatively, within 30 days of ACLR, were used in this study. Most patients (n = 194) had multiple blood pressure assessments (median [IQR]; count = 10 [4]) the day of surgery. For those patients, we compared the first to the average systolic and diastolic blood pressures. The first and the average blood pressures were not significantly different (median [IQR]; systolic blood pressure, 128 [24] vs. 130 [22] mmHg, *p* = 0.21; diastolic blood pressure, 75 [14] vs. 74 [12] mmHg, *p* = 0.73, respectively). The first and the average systolic (r = 0.79, *p* < 0.01, n = 194) and diastolic (r = 0.71, *p* < 0.01, n = 194) blood pressures were also significantly correlated for those patients that had multiple blood pressure recordings the day of ACLR. Based on these results, we decided to use the average systolic and diastolic blood pressure data in the statistical analysis for patients with multiple assessments the day of ACLR.

Total serum 25(OH)D concentrations (sum of D_2_ and D_3_; ng/mL) were determined using a chemiluminescent immunoassay (The Ohio State University Wexner Medical Center, Columbus, OH, USA). Participants were classified as vitamin D-deficient, -insufficient, or -sufficient based on a serum 25(OH)D concentration of ≤20, 21–29, or ≥30 ng/mL, respectively [26]. Seasons were defined as 1 March to 31 May, 1 June to 31 August, 1 September to 31 November, and 1 December to 28 February for spring, summer, fall, and winter, respectively.

### 2.3. Metabolic Syndrome

Patients were separated into the following groups: (1) with MetSy (cases), and (2) without MetSy (controls). Metabolic syndrome components were determined based on the National Cholesterol Education Program Adult Treatment Panel (NCEP ATP) III, with the exception of waist circumference [27]. Waist circumference was not collected as a standard-of-care procedure, and was instead substituted with body mass index (BMI). Body mass index is correlated with waist circumference, and has previously been substituted for waist circumference in other MetSy studies [28,29]. Individuals were defined as having metabolic syndrome if they met any 3 of the following 5 criteria: (1) BMI ≥ 30 kg/m^2^, (2) triglycerides ≥ 150 mg/dL, (3) HDL < 40 mg/dL in men and <50 mg/dL in women, (4) systolic blood pressure ≥ 130 mmHg or diastolic blood pressure ≥ 85 mmHg, or (5) fasting glucose ≥ 100 mg/dL. Fasting glucose was estimated using the following equation [30]:

Estimated fasting glucose (mg/dL) = 28.7 × HbA1c − 46.7

### 2.4. Statistical Analysis

Data were checked for normality with a Shapiro–Wilk test before statistical analysis. Serum 25(OH)D concentrations were log-transformed prior to statistical analyses. Differences in serum 25(OH)D between groups (case vs. control) and between the number of MetSy components were assessed with separate one-way analysis of variance (ANOVA) tests, and, when appropriate, followed by a Bonferroni correction for pairwise comparisons. The season (i.e., winter, spring, summer, and fall) of serum 25(OH)D assessment was used as a statistical co-variate for serum 25(OH)D analyses. Other variable differences were assessed with separate *t*-tests or Mann–Whitney U tests. Fisher’s exact or Chi-Square tests were performed to analyze the associations between categorical variables. Significance was set at *p* < 0.05 and all statistical analyses were performed using SYSTAT (version 13.1, Chicago, IL, USA).

## 3. Results

**Figure 2:**
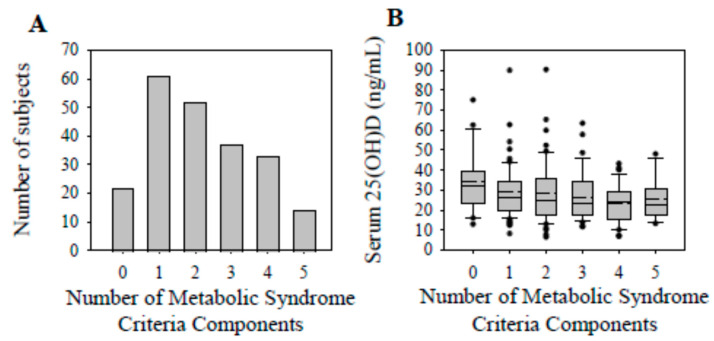
The number of subjects and serum 25(OH)D concentrations (ng/mL) for the number of metabolic syndrome components present following ACLR. (**A**) The number of subjects for the number of metabolic syndrome component counts (i.e., 0 to 5). (**B**) Serum 25(OH)D concentrations based on the number of MetSy components. Serum 25(OH)D concentrations were not significantly different for the number of MetSy components present (*p* = 0.05). Data are presented as box plots (mean, dashed line). Data for all (n = 219 (n = 135 with <3 components; n = 84 with ≥3 components)) patients are included.

The final analysis consisted of 219 patients (median [IQR]: age, 36.7 [15.3] y; BMI, 28.2 [8.6] kg/m^2^; serum 25(OH)D, 25.5 [15.9] ng/mL). Approximately 31.5% (n = 69) of the patients possessed a serum 25(OH)D concentration deemed to classify them as vitamin D-deficient, and ~67% (n = 146) as vitamin D-insufficient or -deficient. The presence of one (27.9%) or two (23.7%) MetSy components was the most common, followed by three (16.9%) and four (15.1%) components (Figure 2A). The presence of zero (10.0%) or all (6.4%) components of MetSy was the least common. 

### 3.1. Patient Characteristics

Metabolic syndrome was found in 38% (n = 84) of the patients (i.e., cases; Table 1). Patient sex, age at ACLR, and height were not significantly different between the cases and controls. Body mass and BMI were significantly increased in the cases (both *p* < 0.01). Approximately 81% of the cases and 18% of the controls were obese based on their BMI (*p* < 0.01). Systolic and diastolic blood pressures were also significantly increased in the cases compared to the controls (both *p* < 0.01).

Total cholesterol and LDL were not significantly different between the cases and controls. However, triglycerides, the cholesterol/HDL-C ratio, and non-HDLC were significantly increased, and HDL was significantly decreased, in cases compared to controls (all *p* < 0.01). The estimated fasting glucose and HbA1c were significantly increased in the cases (both *p* < 0.01). Approximately 28.6% of the cases compared to 11.9% of the controls possessed a HbA1c level of ≥5.7% (*p* < 0.01). The time from ACLR to lipid (median [IQR]; 22.1 [27.7] vs. 23.7 [34.5] months, *p* = 0.43), HbA1c (27.8 [47.6] vs. 30.8 [38.8] months, *p* = 0.28), and serum 25(OH)D (median [IQR]; 29.9 [43.5] vs. 35.0 [54.7] months, *p* = 0.18) assessments were not significantly different between the cases and controls, respectively. The season of serum 25(OH)D assessment was not significantly different between the cases and controls.

### 3.2. Serum 25(OH)D Concentrations and Vitamin D Status

Serum 25(OH)D concentrations reached their nadir (~32% decrease from 0 components) in the presence of 4 components of MetSy (Figure 2B). Regarding the specific components, serum 25(OH)D concentrations were not significantly different with the BMI, blood pressure, or estimated fasting glucose criteria of MetSy (Table 2). In contrast, serum 25(OH)D concentrations were significantly decreased with the triglyceride (*p* = 0.03) and HDL (*p* = 0.02) criteria of MetSy.

**Figure 3:**
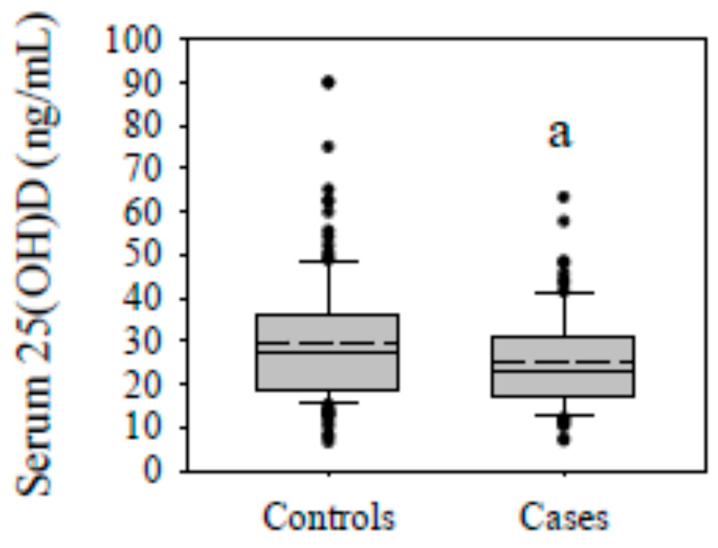
Serum 25(OH)D concentrations (ng/mL) in controls and cases. Serum 25(OH)D concentrations were significantly decreased in cases (with MetSy) compared to controls (without MetSy) after ACLR. Data are presented as box plots (mean, dashed line). ^a^ *p* = 0.01 vs. controls.

Serum 25(OH)D concentrations were 15.8% lower in the cases (mean [SD]; 25.1 [11.3] ng/mL) compared to the controls (29.8 [14.8] ng/mL; *p* < 0.01; Figure 3), and vitamin D sufficiency was found in 26.2% of the cases and 37.8% of the controls (*p* = 0.06; see Table 1). Vitamin D deficiency was found in 13.6%, and sufficiency in 54.6%, of those with no MetSy components (*p* = 0.06; Table 3). The prevalence of vitamin D sufficiency progressively decreased with the increasing presence of one to five (i.e., ~30.1 to 4.1%, respectively; *p* < 0.01) components of MetSy.

## 4. Discussion

The major finding of this study was a decrease in serum 25(OH)D with MetSy following ACLR. In addition, the prevalence of vitamin D sufficiency appeared to decrease with accruing components of MetSy. Serum 25(OH)D concentrations were decreased with the triglyceride and HDL criteria of MetSy. Based on these preliminary findings, MetSy associated with a decrease in serum 25(OH)D and accruing components of this complex syndrome were related to a compromised vitamin D sufficiency status following ACLR.

Sports medicine patients scheduled to undergo surgery (ACLR and/or meniscal repair, including meniscectomy) possess a high prevalence of low serum 25(OH)D [22], and following ACLR, circulating 25(OH)D concentrations decrease [24,25]. The preliminary findings from this investigation extend those observed previously by suggesting a decrease in serum 25(OH)D with MetSy following ACLR. The prevalence of vitamin D sufficiency tended to be greater than that of deficiency in the absence of MetSy components, and there was a progressive decrease in the prevalence of vitamin D sufficiency with an incremental increase in MetSy components following ACLR. With appreciation for the continued uncertainty regarding the actionable 25(OH)D thresholds delineating vitamin D status in relation to different patient-specific outcomes, and the lack of a consensus regarding the concentration demarcating vitamin D deficiency between different organizations or societies [31,32], these data suggest that MetSy and its components are associated with a decrease in serum 25(OH)D and compromised vitamin D sufficiency following ACLR, respectively. However, these findings are limited to post-ACLR blood chemistries, and it is unknown whether pre-surgery assessments and results would be consistent. It is also unknown whether MetSy status and components are altered by ACLR, as this study focused on post-surgical data. Future longitudinal studies consisting of pre- and post-ACLR data examining the relationship between MetSy and vitamin D are warranted.

Consisting of modifiable and interrelated risk factors, MetSy is a conceptual framework linked to the increased development of cardiovascular diseases and type II diabetes mellitus. The early identification and prevention of the first MetSy component may have significant health benefits, as the presence of a single component is predictive in the development of MetSy [5]. Considering that ACL tears frequently occur in a young population [10], patients undergoing and receiving care following an ACL injury and reconstruction could provide a unique opportunity for the early screening of MetSy components, which, in turn, could assist with increasing patient awareness and the development and implementation of diverse treatment strategies intended to protect against or prevent the progression of MetSy and related diseases. Additionally, screening for MetSy in patients that have undergone ACLR could also assist with risk stratification for those with low serum 25(OH)D and provide informative data needed to maximize a supplemental vitamin D intervention strategy.

The potential impact of MetSy on outcomes in patients that have undergone ACLR remains to be fully elucidated, but evidence has emerged regarding the deleterious role of low serum 25(OH)D or a compromised vitamin D status on various outcomes. For example, low serum 25(OH)D has been found to be associated with an increased primary ACL tear rate [33], and after reconstruction, associated with skeletal muscle atrophy and weakness of the involved limb [23,24,34], an increased rate of revision ACLR procedures [33], and a subsequent knee osteoarthritis diagnosis [35]. However, results are inconsistent, as low serum 25(OH)D has been found to not be associated with poor patient reported outcomes or graft failure rates following ACLR [36]. These conflicting results underscore the necessity of future research regarding vitamin D in patients that have suffered an ACL injury and undergone surgery, and the findings here support the need for additional studies exploring the concurrent impact of low serum 25(OH)D and MetSy on outcomes in patients that have undergone ACLR.

Although further research is needed to identify whether MetSy following ACLR is associated with cardiovascular diseases, diabetes, and other illnesses, diseases, and complications, MetSy has been found to be associated with knee osteoarthritis [7,8], a joint disease that commonly follows an ACL injury and surgery. Among other characteristics, a loss of cartilage volume and changes in bone marrow lesions are hallmarks of knee osteoarthritis [37]. In older patients, MetSy is an independent risk factor for greater medial tibial cartilage volume loss, and the presence of bone marrow lesions in the knee joint is more prevalent with MetSy [7]. Medial tibial cartilage volume loss and the proportion of participants displaying a substantial bone marrow lesion size (i.e., >52 mm^2^) have been found to increase with the number of MetSy components present [7]. These data appear to suggest that there is an increase in the onset and progression of knee osteoarthritis with an increase in the number of MetSy components [7,8].

Parallel to the number of MetSy components are mechanistic pathways that contribute to the pathophysiology of MetSy. Adipose tissue dysfunction is a regulatory and etiological factor of MetSy that potentially contributes to impairment in extracellular matrix remodeling, immune cell infiltration, and altered pro-/anti-inflammatory cytokine secretion and balance [38,39,40]. The cardiometabolic risk posed by MetSy is associated with insulin resistance, which, among other factors, affects glucose metabolism and the production of reactive oxygen and nitrogen species that promote inflammation and impair cellular metabolism [38,39,40]. Both adipose tissue dysfunction and insulin resistance contribute to inflammation, and reciprocally, chronic, low-grade inflammation with MetSy has been demonstrated to disrupt adipose tissue function and potentially contribute to insulin resistance [38,39,40]. Other factors also contribute to the pathophysiology of MetSy, highlighting the need for additional research examining various signaling pathways and mechanisms related to outcomes following ACLR.

Circulating 25(OH)D is regulated by a variety of endogenous and exogenous factors. Although identifying the mechanism or mechanisms mediating the decrease in serum 25(OH)D with MetSy following ACLR was not the intent of this study, serum 25(OH)D concentrations were lower with the triglyceride and HDL criterion of MetSy. These data are consistent with other results demonstrating an inverse association between serum 25(OH)D and triglycerides and a positive association between serum 25(OH)D and HDL [12,41,42,43,44]. Extrapolating from experimental animal data, it is plausible that circulating triglyceride and HDL levels are altered by a high-fat diet and mediate a decrease in serum 25(OH)D by suppressing the hepatic expression of CYP2R1 [45], a 25-hydroxylase essential in the conversion of vitamin D to 25(OH)D. However, it is also reasonable that other mechanisms contribute to the decrease in serum 25(OH)D with MetSy. For example, dietary, environmental, genetic, inflammation, insulin resistance, adiposity, and peri-operative procedures are only a few plausible pathways that could govern a decrease in serum 25(OH)D with MetSy following ACLR that were not accounted for in the present study. Nonetheless, while the data suggest an association between lipids and circulating 25(OH)D, additional research examining the role of MetSy and its components in the regulation of circulating 25(OH)D concentrations following ACLR is required, along with the concurrent consideration of other regulators of circulating 25(OH)D (e.g., dietary intake of vitamin D, genetics, inflammation, skin pigmentation, physical activity, and sun exposure) [46]. Additional research should also account for antihypertensive, lipid-lowering, and antidiabetic medications and the use of supplemental vitamin D.

In addition to those discussed above, there are other limitations to this study that are worthy of discussion. This study consisted of a retrospective study design and was limited to a relatively small sample size of patients with available data in the electronic medical records. Due to these limitations, it is plausible the prevalence of MetSy and its components were overestimated, potentially due to selection bias and data completeness requirements. This approach potentially introduced selection bias by including patients with suspected metabolic abnormalities and excluding patients without complete data. The retrospective design also limited the ability to account for various confounders, such as vitamin D supplementation and sunlight exposure. Also, modified MetSy criteria were implemented in this study, as waist circumference and fasting glucose were not consistently available in the electronic medical records. As such, waist circumference was substituted with BMI, and fasting glucose was estimated using HbA1c. Substituting BMI for waist circumference has been found to produce similar results when investigating the association between serum 25(OH)D and MetSy [47]. In the future, comparing serum 25(OH)D concentrations between ACLR patients and matched, non-ACLR patients with and without MetSy would advance our knowledge regarding the relationship between MetSy and vitamin D in this patient population. This study also lacks longitudinal and long-term follow-up data regarding the association between MetSy and vitamin D, and the possibility of reverse causation, or of low vitamin D contributing to MetSy following ACLR [48], warrants future investigation.

## 5. Conclusions

Identifying the components of MetSy in young cohorts could assist with early screening and treatments intended to reduce the incidence of this complex syndrome and related diseases. Based on the preliminary nature of the findings here, we conclude that accruing components and evidence of MetSy could be detrimental to serum 25(OH)D concentrations following ACLR. However, prospective studies initiated prior to and continued after ACLR that utilize larger sample sizes, and that take into consideration potential confounding variables of vitamin D and recognize ACL injury mechanisms (and the time from injury to surgery), are encouraged.

## Figures and Tables

**Table 1:** Patient characteristics for controls (without MetSy) and cases (with MetSy).

	Controls	Cases	*p*-Value
n (f/m)	135 (83/52)	84 (43/41)	0.16 *
Age, y	37.4 (15.1)	35.5 (15.7)	0.55
Height, m	1.70 (0.14)	1.70 (0.15)	0.32
Body Mass, kg	75.3 (23.6)	99.8 (27.6)	<0.01
BMI, kg/m^2^	26.0 (6.0)	33.9 (8.6)	<0.01
BMI Classifications			<0.01
Underweight (≤18.5 kg/m^2^), n (%)	2 (1.5)	0	
Healthy Weight (18.5–24.9 kg/m^2^), n (%)	52 (38.5)	8 (9.5)	
Overweight (25.0–29.9 kg/m^2^), n (%)	57 (42.2)	8 (9.5)	
Obese (30.0–39.9 kg/m^2^), n (%)	21 (15.6)	49 (58.3)	
Severe Obesity (≥40.0 kg/m^2^), n (%)	3 (2.2)	19 (22.6)	
Systolic Blood Pressure, mmHg	123 (19)	138 (16)	<0.01
Diastolic Blood Pressure, mmHg	72 (12)	78 (10)	<0.01
Cholesterol, mg/dL	184 (43)	193 (45)	0.27
Triglycerides, mg/dL	83.0 (46.5)	168 (132)	<0.01
HDL, mg/dL	56.0 (21.8)	43.0 (130)	<0.01
LDL, mg/dL	103 (49)	103 (43)	0.46
Cholesterol/HDL-C Ratio	3.05 (1.30)	4.20 (1.40)	<0.01
Non-HDLC, mg/dL	124 (51)	140 (40)	<0.01
HbA1c, %	5.20 (0.50)	5.50 (0.40)	<0.01
HbA1c Classification			<0.01
Normal (<5.7%), n (%)	119 (88.2)	60 (71.4)	
Prediabetic (5.7–6.4%), n (%)	15 (11.1)	17 (20.2)	
Diabetic (>6.4%), n (%)	1 (0.7)	7 (8.3)	
Estimated Fasting Glucose, mg/dL	103 (14)	111 (11)	<0.01
Vitamin D Status			0.06
Deficient, n (%)	35 (25.9)	34 (40.5)	
Insufficient, n (%)	49 (36.3)	28 (33.3)	
Sufficient, n (%)	51 (37.8)	22 (26.2)	
Season of 25(OH)D Assessment			0.54
Fall, n (%)	33 (24.4)	24 (28.6)	
Winter, n (%)	29 (21.5)	21 (25.0)	
Spring, n (%)	34 (25.2)	22 (26.2)	
Summer, n (%)	39 (28.9)	17 (20.2)	

Data are presented as median (IQR) and n (%), unless noted otherwise. * *p*-value, sex count (n) comparison between controls and cases.

**Table 2:** Serum 25(OH)D concentrations separated based on the individual MetSy criteria for all subjects.

		Serum 25(OH)D (ng/mL)	*p*-Value *
BMI			0.26
	<30 kg/m^2^ (n = 127)	26.0 (16.1)	
	≥30 kg/m^2^ (n = 92)	24.0 (15.8)	
Triglycerides			0.03
	<150 mg/dL (n = 156)	26.5 (17.5)	
	≥150 mg/dL (n = 63)	23.6 (12.7)	
HDL			0.02
	males ≥ 40 and females ≥ 50 mg/dL (n = 154)	25.7 (17.3)	
	males < 40 and females < 50 mg/dL (n = 65)	25.0 (13.2)	
Blood pressure			0.11
	SBP < 130 and DBP < 85 mmHg (n = 107)	27.4 (17.4)	
	SBP ≥ 130 or DBP ≥ 85 mmHg (n = 112)	23.5 (14.9)	
Estimated fasting glucose			0.13
	<100 mg/dL (n = 73)	28.2 (13.3)	
	≥100 mg/dL (n = 146)	23.8 (16.0)	

Serum 25(OH)D concentration data, presented as the median (IQR), for each individual component of MetSy, and the number of subjects that met the criteria for each component. * *p*-value of serum 25(OH)D concentration comparisons for individual metabolic syndrome criteria components. DBP, diastolic blood pressure; SBP, systolic blood pressure; n = 219.

**Table 3:** The association between the number of MetSy components and vitamin D status.

Number of MetSy Components	Vitamin D Status			Row *p*-Value
	Deficient	Insufficient	Sufficient	
	n = 69	n = 77	n = 73	
0, n = 22 (row %)	3 (13.6)	7 (31.8)	12 (54.6)	0.06
(column %)	(4.4)	(9.1)	(16.4)	
1, n = 61 (row %)	15 (24.6)	24 (39.3)	22 (36.1)	0.33
(column %)	(21.7)	(31.2)	(30.1)	
2, n = 52 (row %)	17 (32.7)	18 (34.6)	17 (32.7)	0.98
(column %)	(24.6)	(23.4)	(23.3)	
3, n = 37 (row %)	16 (43.2)	9 (24.3)	12 (32.4)	0.37
(column %)	(23.2)	(11.7)	(16.4)	
4, n = 33 (row %)	13 (39.4)	13 (39.4)	7 (21.2)	0.34
(column %)	(18.8)	(16.9)	(9.6)	
5, n = 14 (row %)	5 (35.7)	6 (42.9)	3 (21.4)	0.61
(column %)	(7.3)	(7.8)	(4.1)	
Column *p*-Value	<0.01	<0.01	<0.01	

Data are presented as n (%). The rows represent the number of subjects (%) for a given number of MetSy components (i.e., 0, 1, 2, 3, 4, 5) in relation to vitamin D status, with the corresponding *p*-value provided for each row. For example, twenty-two subjects possessed zero components of MetSy. Of those subjects, vitamin D deficiency was found in 3, insufficiency in 7, and sufficiency in 12 (*p* = 0.06). The columns represent the number of subjects (%) for a given vitamin D status (i.e., Deficient, Insufficient, Sufficient) in relation to the number of MetSy components, with the *p*-value provided at the bottom of the corresponding column. Significance was assessed with Chi-Square tests.

## Data Availability

The data underlying this article will be shared upon reasonable request to the corresponding author due to privacy.
